# Contingency and congruency switch in the congruency sequence effect: a reply to Blais, Stefanidi, and Brewer (2014)

**DOI:** 10.3389/fpsyg.2014.01405

**Published:** 2014-12-09

**Authors:** James R. Schmidt

**Affiliations:** Department of Experimental Clinical and Health Psychology, Ghent UniversityGhent, Belgium

**Keywords:** congruency sequence effect, Gratton effect, contingency learning, switch costs, conflict adaptation, cognitive control, attention, feature integration

## Introduction

The congruency sequence effect (CSE) is the observation that the congruency effect is reduced following an incongruent trial (Gratton et al., 1992). Generally, the CSE is interpreted in terms of *conflict adaptation*, the idea that participants decrease attention to the distracter and/or increase attention to the target after experiencing conflict (e.g., Botvinick et al., 2001). An alternative *learning and memory* account proposes that the CSE is instead due to basic learning confounds (for a review, see Schmidt, 2013). For instance, systematic differences in the types of feature repetitions that are possible in each cell of the design might produce a CSE (Mayr et al., 2003; Hommel et al., 2004). Schmidt and De Houwer (2011) considered two additional learning and memory biases: sequential contingencies and congruency switch costs. However, Blais et al. (2014) present data which they suggest argue against a role of these two biases. This article illustrates some issues with this work and suggests that contingency and congruency switch biases may play a role after all.

## Contingency analysis underpowered

Schmidt and De Houwer (2011) introduced the idea that *sequential contingency* biases might influence the CSE. Often, each distracter is presented more frequently in the congruent color than in each incongruent color. Unfortunately, this introduces a contingency, whereby words are predictive of the congruent response. Contingency biases are larger following an accurately predictive trial than following an incorrectly predictive trial (Schmidt et al., 2007). Thus, contingency biases can contribute to the CSE. Indeed, Mordkoff (2012) showed that, with feature repetitions removed, the CSE *is* present in a contingency-biased Simon task, but *is not* observed in a contingency-unbiased version of the same task.

Blais et al. (2014) report a reanalysis of verbal Stroop data in which each participant performed several blocks of trials with varying contingencies (as manipulated with proportions of congruent trials) from 5 to 95% in increments of 5% (though only 10 to 80% could be analyzed). Overall, CSEs were not reliable for most contingency levels. Critically, the CSE did not significantly increase as a function of contingency in response times. With these data, the authors argued that contingencies are unlikely to play a role in the CSE.

**Figure 1:**
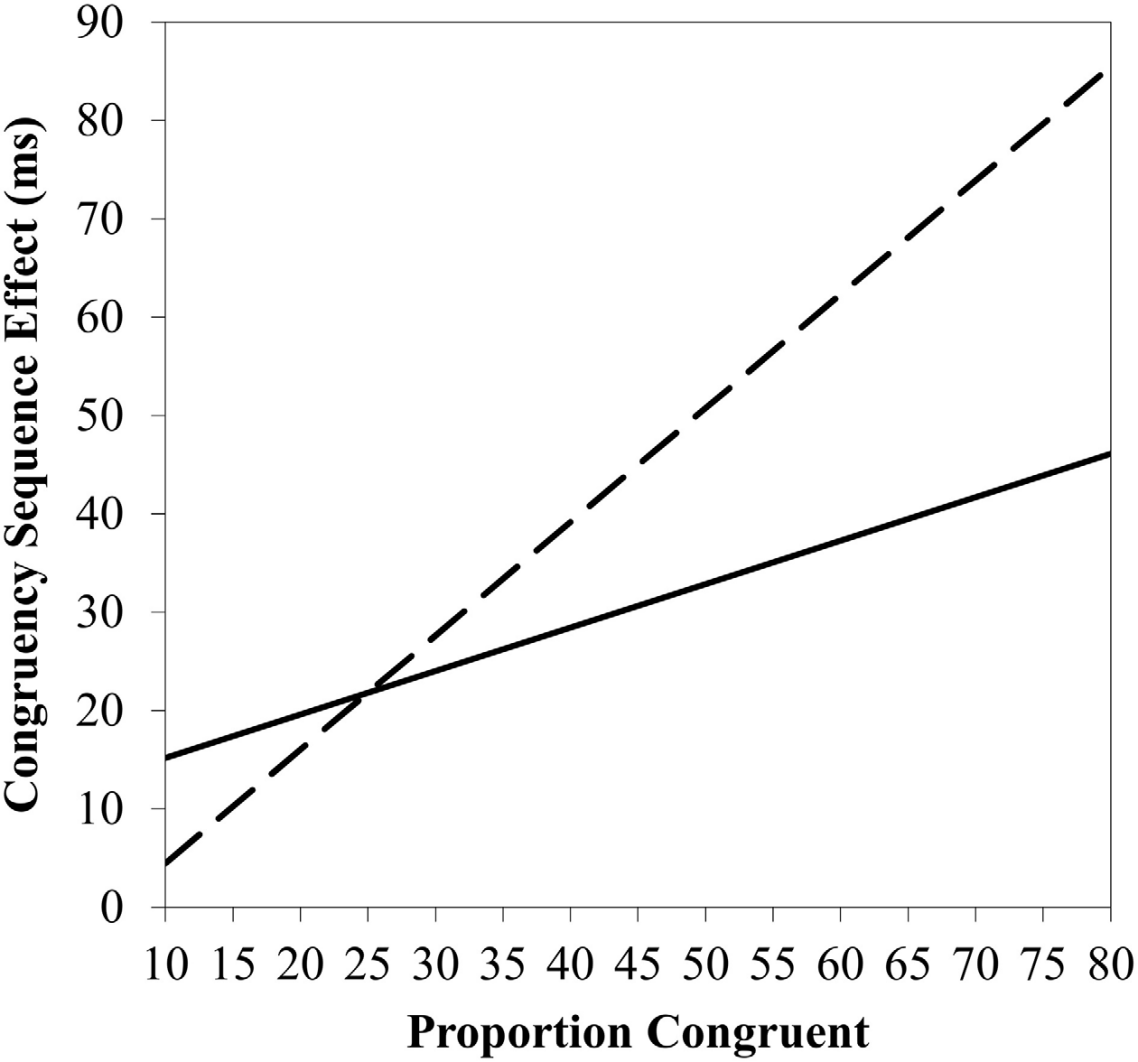
**Congruence sequence effect as a function of proportion congruency, with observed trend line (solid line) and trend line that would have been required for a high power test given the sample size and error (dashed line)**.

However, statistical power of the sample of 15 participants is a concern. Indeed, the slope was notably positive, but with considerable error, *B* = 0.438 ± 0.382, *t*_(14)_ = 1.15, *p* > 0.25. The *B* parameter is the amount of change in the CSE for a 1% contingency increment (i.e., 2.2 ms for each 5% increment, and 31 ms overall). Though not significant, this represents a medium effect size (β = 0.315). As Figure 1 illustrates, the study only had high (0.8) power to detect a large effect size (β = 0.661). As a further concern, contingencies were manipulated between blocks. Contingency biases are known to transfer across blocks (Schmidt et al., 2010), causing contamination. Thus, contingency biases were probably underestimated. Curiously, the slope for the errors *was* significant and in the predicted direction, *B* = 0.038 ± 0.017, *t*_(14)_ = 2.26, *p* = 0.040. Though seemingly confirming a role of contingencies, the authors reasoned that this slope is difficult to interpret given that none of the CSE estimates for the various contingency levels were significant. This argument does not seem particularly convincing, only feeding concerns about statistical power.

## Congruency switch hypothesis, revised

Schmidt and De Houwer (2011) further considered the possibility that there might be encoding costs associated with “switching” from a congruent to an incongruent trial, or vice versa, relative to repeating the same type of trial. Thus, following an incongruent trial, incongruent trials will incur a benefit and congruent trials a loss. The reverse is true following a congruent trial. As a result, *congruency switch costs* can further explain variance in the CSE.

Schmidt and De Houwer (2011) suggested that the cost of switching from congruent to incongruent might be “roughly” the same as the reverse, but Blais et al. (2014) did not observe this additivity. In retrospect, this was a misguided prediction. It is known that switching from a hard (non-dominant) to an easy (dominant) task sometimes incurs a larger cost on performance than the reverse, known as *switch cost asymmetry* (see Allport et al., 1994). The same might be predicted here, where a congruent “encoding shortcut” might be especially fast following a congruent trial, whereas the harder encoding task on incongruent trials will take long regardless of the previous trial congruency. This is an intriguing suggestion, because the conflict adaptation account should predict the exact opposite: because Stroop effects are primarily interference driven (see MacLeod, 1991), changes in attention to the word should be reflected primarily in incongruent trials.

Looking closely at the data of Blais et al. (2014), it can be seen that the interaction between congruency and congruency switch is due entirely to a larger effect of congruency switch for congruent trials (Experiment 1: 32 ms; Experiment 2: 44 ms) than for incongruent trials (Experiment 1: −4 ms; Experiment 2: −15 ms). Thus, this interaction is inconsistent with the conflict adaptation account, but is consistent with a revised version of the congruency switch hypothesis.

## Conclusion

Though the current paper does not contest the notion that CSEs can be observed independent of feature repetition and contingency learning biases (e.g., Kim and Cho, 2014; Schmidt and Weissman, 2014; Weissman et al., 2014), three inferences of Blais et al. (2014) are contestable. First, contingency biases probably do play a role in the effect, as indicated by the significant effect in the errors, the underpowered but notable trend in the response times, and the data of Mordkoff (2012). Second, congruency switch effects might also play a role, as indicated by the direction of the switch cost asymmetry. Third, Blais and colleagues too quickly attribute the remaining CSEs to conflict adaptation. Yet other accounts still remain, such as the temporal learning and activation-suppression accounts, which actually seem to fit the extant data better than the conflict adaptation account (e.g., see Weissman et al., 2014, Schmidt and Weissman, in review).

### Conflict of interest statement

The Associate Review Editor Wim Notebaert declares that, despite being affiliated to the same institution as the author James R. Schmidt, the review process was handled objectively and no conflict of interest exists. The author declares that the research was conducted in the absence of any commercial or financial relationships that could be construed as a potential conflict of interest.
